# Whole genome sequence comparison of *vtx2*-converting phages from Enteroaggregative Haemorrhagic *Escherichia coli* strains

**DOI:** 10.1186/1471-2164-15-574

**Published:** 2014-07-08

**Authors:** Laura Grande, Valeria Michelacci, Rosangela Tozzoli, Paola Ranieri, Antonella Maugliani, Alfredo Caprioli, Stefano Morabito

**Affiliations:** EU Reference Laboratory for E. coli, Veterinary Public Health and Food Safety Department, Istituto Superiore di Sanità, Viale Regina Elena 299, Rome, 00161 Italy; Università degli Studi di Roma Tre, Rome, Italy

**Keywords:** Enteroaggregative haemorrhagic *E. coli*, *vtx*-phages, Whole genome sequence, Tail fibers

## Abstract

**Background:**

Enteroaggregative Haemorrhagic *E. coli* (EAHEC) is a new pathogenic group of *E. coli* characterized by the presence of a *vtx2*-phage integrated in the genomic backbone of Enteroaggregative *E. coli* (EAggEC). So far, four distinct EAHEC serotypes have been described that caused, beside the large outbreak of infection occurred in Germany in 2011, a small outbreak and six sporadic cases of HUS in the time span 1992–2012. In the present work we determined the whole genome sequence of the *vtx2*-phage, termed Phi-191, present in the first described EAHEC O111:H2 isolated in France in 1992 and compared it with those of the *vtx*-phages whose sequences were available.

**Results:**

The whole genome sequence of the Phi-191 phage was identical to that of the *vtx2*-phage P13374 present in the EAHEC O104:H4 strain isolated during the German outbreak 20 years later. Moreover, it was also almost identical to those of the other *vtx*2-phages of EAHEC O104:H4 strains described so far. Conversely, the Phi-191 phage appeared to be different from the *vtx*2-phage carried by the EAHEC O111:H21 isolated in the Northern Ireland in 2012.

The comparison of the *vtx2*-phages sequences from EAHEC strains with those from the *vtx*-phages of typical Verocytotoxin-producing *E. coli* strains showed the presence of a 900 bp sequence uniquely associated with EAHEC phages and encoding a tail fiber.

**Conclusions:**

At least two different *vtx2*-phages, both characterized by the presence of a peculiar tail fiber-coding gene, intervened in the emergence of EAHEC. The finding of an identical *vtx2*-phage in two EAggEC strains isolated after 20 years in spite of the high variability described for *vtx*-phages is unexpected and suggests that such *vtx2*-phages are kept under a strong selective pressure.

The observation that different EAHEC infections have been traced back to countries where EAggEC infections are endemic and the treatment of human sewage is often ineffective suggests that such countries may represent the cradle for the emergence of the EAHEC pathotype. In these regions, EAggEC of human origin can extensively contaminate the environment where they can meet free *vtx*-phages likely spread by ruminants excreta.

**Electronic supplementary material:**

The online version of this article (doi:10.1186/1471-2164-15-574) contains supplementary material, which is available to authorized users.

## Background

Diarrheagenic *Escherichia coli* (DEC) are a heterogeneous group of pathogenic *E. coli* causing a wide range of enteric diseases in humans and animals [1].

Enteroaggregative *E. coli* (EAggEC) are a DEC pathotype inducing a gastrointestinal illness characterized by long-lasting watery, mucoid, secretory diarrhoea with low-grade fever and little or no vomiting [2, 3]. EAggEC infections are a common cause of acute diarrheal illness among children in low-income countries, but sporadic cases and outbreaks are recorded in industrialized countries as well [4, 5].

In 2011, a large *E. coli* outbreak struck Germany causing more than 4,000 human infections including 50 deaths [6]. The outbreak strain, an *E. coli* O104:H4, showed the presence of the typical virulence genes of EAggEC such as *aggR*, *aaiC*, *sepA*, *aatA* and, at the same time, it carried a bacteriophage conveying the genes encoding the Verocytotoxin (Vtx) subtype 2a (*vtx2a*) [7]. In accordance with this genomic asset, the strain showed the Enteroaggregative typical “stacked brick” adhesion to cultured Hep-2 cells and was able to produce Vtx2 [8]. The infecting strain thus displayed an unusual combination of virulence features comprising the colonization repertoire from EAggEC coupled with the production of a toxin typically produced by Vtx-producing *E. coli* (VTEC), a DEC type causing haemorrhagic colitis and Haemolytic Uremic Syndrome (HUS) worldwide [1].

The impact of the German outbreak was so huge that the epidemic strain became iconic of a new DEC type: the Enteroaggregative Haemorrhagic *E. coli* (EAHEC) [9]. The occurrence of the German outbreak also caused the scientific community to look retrospectively at the reported HUS cases linked to infections with atypical VTEC types or to browse the scientific literature in order to assess if other EAHEC cases of infection could be retrieved. It turned out that in the time period 1992–2012 a small outbreak and at least six sporadic cases of HUS had been described as being associated with EAHEC strains belonging to four different serotypes: O111:H2, O86: HNM, O104:H4 and O111:H21 [8, 10–13].

The analysis of the whole genome sequence of the EAHEC O104:H4 that caused the German outbreak in 2011 showed that the *vtx2*-phage is inserted in a bacterial genomic backbone typical of EAggEC [14], therefore the EAHEC pathotype seems to have arisen from the acquisition of *vtx2*-phages by classical EAggEC strains.

The appearance of the EAHEC group has shown that the stable acquisition of *vtx*-phages seems to have occurred at least twice by two different DEC groups, the EAggEC and the atypical EPEC (aEPEC) from which the typical VTEC pathotype derives [15–17]. Moreover, the ability of *vtx2*-phages to infect, in the laboratory conditions, different *E. coli* pathogroups including ExPEC has been reported [18, 19]. This observation, together with the isolation of Enterobacteriaceae other than *E. coli* producing Vtx from cases of human disease [20, 21] suggests that *vtx*-phages can infect a range of bacterial hosts wider than expected, confirming the pivotal role of phages in the evolution of bacterial pathogens.

In the present work we determined the whole genome sequence of the *vtx2*-phage present in the first EAHEC ever described and compared it with that of the *vtx2*-phages present in the EAHEC O104:H4 and O111:H21 available in the public repositories and with those of other *vtx*-phages, with the aim of investigating the mechanisms underlying the evolution of the EAHEC pathotype.

## Methods

### Bacterial strains

The EAHEC O111:H2 strain ED 191 has been used to obtain the *vtx2*-phage subjected to whole genome sequencing and is part of the collections held at Istituto Superiore di Sanità. The strain’s characteristics have been described in a previous publication [10].

*E. coli* K12 strain LE392 [22] has been used as a propagator strain in infection experiments for the *vtx2*-phage amplification prior to sequencing.

### Determination of the vtx2-phages integration sites in the E. coli genome

The *vtx2*-phage integration site in the *E. coli* strain ED 191 has been determined. The occupancy of loci *sbcB, wrbA, yehV*, Z2577, and *yecE* has been assessed as previously described [18].

### Infection experiments and phages propagation

The EAHEC strain ED 191 has been exposed to UV light in order to induce the excision of phage genome from the bacterial chromosome [23]. In detail, the bacterial strain has been grown in Luria-Bertrani (LB) broth (Oxoid Limited, Basingstoke Hampshire, UK) overnight at 37°C with vigorous shaking. The culture has been diluted 1:100 in LB modified broth (LB with 0.001% thiamine V/V) and grown to 0.5 OD 600, pelleted and re-suspended in a sterile solution of CaCl_2_ 10 mM. The culture has been exposed to UV light (130 μJoule X 100) in a crosslinker “Stratalinker® UV crosslinker” (Stratagene Cloning Systems, La Jolla, CA, USA). After induction, the culture has been diluted in LB modified broth and incubated at 37°C for 5 hours with vigorous shaking. The culture has been centrifuged and the supernatant containing phages particles filtered with 0,22 μm pore-filters. 100 μl of phage particles suspension have been added to 100 μl of a culture of the propagator strain *E. coli* LE392 grown in LB modified broth at 0.5 OD 600 and maintained at 37°C for 20 minutes with static incubation. Each tube has been added with 3.5 ml of LB modified soft agar (LB modified broth with agar 7 g/L) at 42°C and immediately poured on LB modified agar plates (LB modified broth with 15 g/L agar). Plates have been incubated overnight at 37°C.

Four ml of SM buffer (100 mM NaCl, 8 mM MgSO_4_·7H_2_O, 50 mM Tris–HCl 1 M pH 7.5, Gelatin 0.002%) have been dispensed to each plate in order to recover phages particles from the lytic plaques and kept overnight at 4°C. The phage suspension in SM has been recovered and chloroform has been added at 5% final concentration. The phage suspension has been centrifuged at 500*xg* 10 minutes twice for removing agar debris and used to re-infect the propagator *E. coli* strain LE392 in the conditions described above in order to increase the phage titre. Finally, the phage suspension has been concentrated by using Amicon Ultra-15 Centrifugal Filter Unit with Ultracel-30 tubes (Merck Millipore, Billerica, MA, USA) with a cut-off of 30 KDa. Final phage titre was 7 × 10^10^ PFU/ml.

### CsCl gradient and viral DNA extraction

The suspension has been purified by Isopycnic Centrifugation through CsCl Equilibrium gradient as described by Sambrook and Russell [23]. Briefly, 2 ml of the phage suspension have been added with 1.5 g of CsCl, transferred to ultracentrifuge tubes, which have been filled with a CsCl solution 0.75 g/ml. The tubes have been finally sealed with mineral oil and centrifuged in a Beckman ultracentrifuge at 154,000*xg*, 8°C for 20 hours in a SW-41 rotor. The band containing the phage particles has been collected with a syringe by puncturing the tube. The recovered solution containing the purified phage particles has been dialyzed in against 10 mM NaCl, 50 mM Tris–HCl pH 8.0, 10 mM MgCl_2_. A final volume of 1 ml was obtained.

The suspension has been treated by adding 100 units of DNase I RNase-free (New England Biolabs, USA) at 37°C for one hour to eliminate free DNA contaminating the phage suspension. Finally a treatment with proteinase K 50 μg/ml at 56°C for one hour has been carried out to disrupt the phage capsid followed by DNA extraction with phenol-chloroform-isoamyl alcohol [23]. Phage DNA concentration after the purification step was estimated to be 239.4 ng/μl.

### Library preparation and whole genome sequencing of the phage DNA

Phage DNA has been sequenced with an Ion Torrent PGM semiconductor sequencer (Life Technologies, Carlsbad, USA) using the 200 bp protocol. An Ion Torrent 314 chip has been used following the manufacturer instructions (Life Technologies, Carlsbad, USA). Genomic library has been obtained by shearing 1 μg of DNA in blunt-ended fragments followed by linking the Ion Adapters using the protocol included in the Ion Xpress™ Plus Fragment Library Kit (Life Technologies, Carlsbad, USA). The sized and ligated fragments have been amplified by emulsion-PCR using the Ion OneTouch 200 Template kit and instruments (Life Technologies, Carlsbad, USA).

### Assembly and further bioinformatics analyses

The reads resulting from the sequencing of the *vtx2*-phage DNA from the EAHEC O111:H2 strain, termed Phi-191, have been assembled in contigs by using the open source MIRA software integrated in the Ion Torrent Server. Contigs have been imported in Kodon software (Applied Maths NV, Sint-Martens-Latem, BE) for analysis. To fill in the gaps between contigs, a total of 95 primers have been designed and used for sequencing by Sanger technology using a Genetic analyzer 3130 (Life Technologies, Carlsbad, USA). Mauve software [24] has been used to order the contigs using the sequence of the phage P13374 from the *E. coli* O104:H4 that caused the outbreak in Germany in 2011 [GenBank: NC_018846.1] as reference. The complete sequence of the Phi-191 phage has been annotated by Prokka tool on the online server Galaxy/CRS4 [25] and submitted to GenBank [GenBank: KF971864]. The G + C content has been analysed by the GC calculator free online tool [26]. Identification of putative tRNA genes has been performed using tRNAscan-SE [27].

The raw sequence data (short reads) from the EAHEC O111:H21 strain 226 were retrieved from the SRA database present on NCBI website [NCBI SRA: SRA055981] and aligned on the complete sequence of Phi-191 phage, determined in the present study and used as reference, with the Bowtie2 free software implemented in the Galaxy/CRS4 server [25].

Genomic comparisons between the available *vtx*-phages sequences have been performed by using the BLAST algorithm available at NCBI [28] and the Mauve free alignment software [24]. Comparison map between *vtx*-phages has been generated by Circoletto online tool [29, 30]. For the pictogram construction, bit-score values have been used to describe the quality of the alignment at a given point. The bit-score is a normalized version of the score value returned by the BLAST searches, expressed in bits [28].

### Ethics

This work does not include animal testing and does not report human data. All the information regarding outbreaks and cases of infection are all from already published papers properly referenced in the text.

## Results

### Sequencing of the vtx2-phage from the EAHEC O111:H2

The whole genome sequencing of the *vtx2-*phage from the EAHEC O111:H2 strain, Phi-191, produced 320,044 reads of a mean length of 204 bp, for a total of 65.30 Mb sequenced. The assembly of the Phi-191 sequence reads using as a reference the genome of the P13374 phage [GenBank: NC_018846.1] produced 151 contigs that were further analysed using bioinformatics resources. The estimated coverage of the phage genome was 1088X.

**Figure 1:**
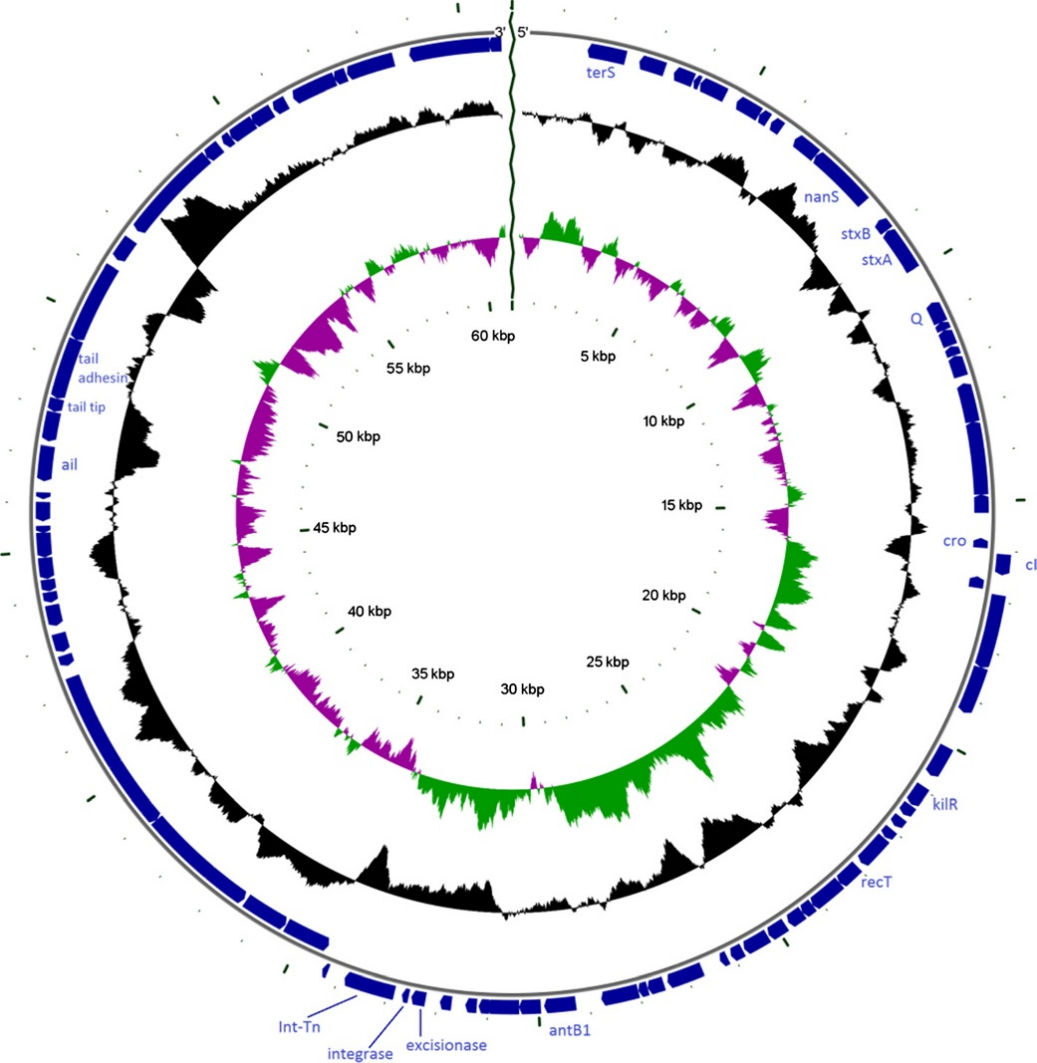
**Genomic organization of Phi-191 phage.** Coding sequences are represented as blue bars in the outer circle. Putative genes are labelled according to the predicted functions of their products, if known. The whole GC content and GC skew of leading and lagging strand is shown in the black and coloured inner circles, respectively.

The length of the whole sequence of Phi-191 resulted to be about 61 Kb (61,036 bp) [GenBank: KF971864] with a mean G + C content of 50.2%.Sequence annotation revealed the presence of 87 predicted coding sequences including the genes encoding the subtype 2a of the Verocytotoxin and three transfer RNAs (tRNAs) (Figure 1).

As it has been reported for the P13374 phage, conveying the *vtx2* genes in the EAHEC O104:H4 strain that caused the German outbreak in 2011 [31], the genome of Phi-191 (Figure 1) only included the lambda genes *cI* and *cro* while the other genes typically composing the regulatory repertoire of lambda phages such as *cII*, *cIII, N, Ea10* and *gam* seemed not to be present [31]. Most of these genes are involved in the regulation of the switch between lysogeny and lytic cycle in lambda-like phages. Therefore, their absence suggests the existence of an alternative mechanism used to regulate the choice of entry into lysogenic state. The Phi-191 phage is integrated in the bacterial chromosome in the *wrbA* locus, as it has been described for P13374 [31].

### Comparison of the Phi-191 whole genome sequence with other vtx-phage sequences

A BLAST search of the Phi-191 *vtx2-*phage whole genome sequence against those collected in the nucleotide repository held at NCBI, returned a 99% identity with that of the P13374 phage from O104:H4 strain CB13374 which caused the German outbreak in 2011 [GenBank:NC_018846.1] and a 91% identity with that of the *vtx2*-phage TL-2011c carried by the VTEC O103:H25 strain that caused a severe HUS outbreak in Norway in 2006 [GenBank:JQ011318] [32]. This finding is in agreement with how reported in the literature for the *vtx*-phage carried by the EAHEC O104:H4 from the German outbreak and the TL-2011c phage [33]. As expected, the Phi-191 phage sequence also showed 99% identity with the *vtx2*-phage from another EAHEC O104:H4 strain isolated during the 2011 German outbreak, the strain 2011C-3493 [GenBank:CP0032891.1] and was highly related (97% nucleotide sequence identity) to the sequence of two *vtx*2-phages from two EAHEC O104:H4 strains isolated from as many haemorrhagic colitis cases occurred in Georgia in 2009 [GenBank:CP003301.1, CP003297.1] [34].

**Figure 2:**
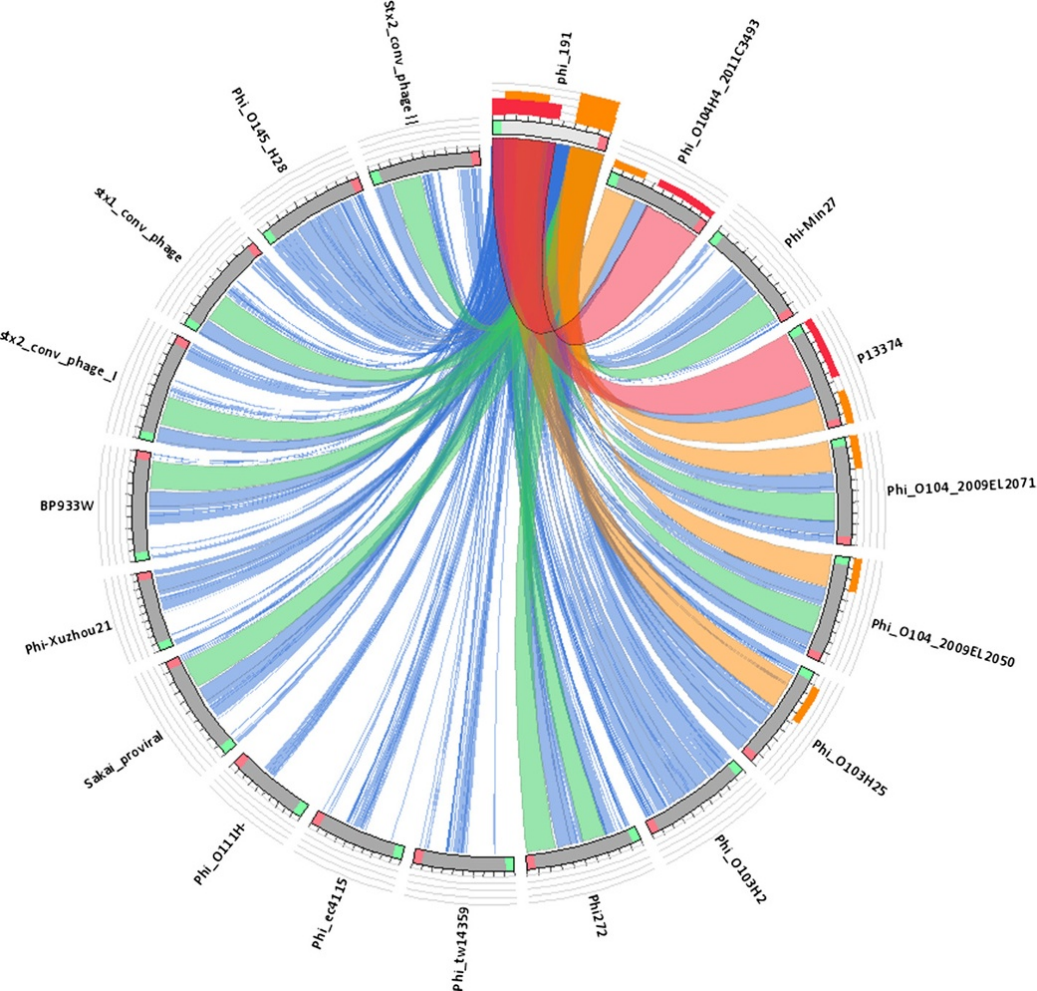
**Sequence similarities between Phi-191 and other** ***vtx*** **-phages.** The picture shows the results of the BLAST local alignments using Phi-191 as a query against the *vtx*-phage sequences with 99% to 60% similarity listed in Table 1. The colours codes blue, green, orange and red represent the overall quality of the aligned segments along the phage sequences, evaluated on the basis of the bit-score values in the worst-to-the-best order (blue to red). The bit-score is a normalized version of the score value returned by the BLAST searches, expressed in bits [28]. The height of the coloured bars in the histogram on the top of the Phi-191 ideogram shows how many times each colour hits a specific fragment of the other phage sequences. A twist in a ribbon indicates that the local alignment is inverted (query and database sequence on opposite strands).

The other scores included hits with query coverage values ranging from 87% down to 60% and having 98%-99% sequence identity, with a number of other *vtx-*phages identified in different VTEC strains (Table 1). The alignment of all the phage sequences comprised in the 100%-60% similarity range is shown in a pictogram generated with the Circoletto online tool [30] (Figure 2) and in another one produced by Mauve software (in Additional file 1: Figure S1).

**Table 1:** **List of the BLAST hits of Phi-191 DNA sequence aligned to the** ***vtx*** **-phages sequences from typical VTEC strains**

Strain	Similarity%	Acc. No.	Reference
*E. coli* O145:H28 str. RM13514	87%	CP006027.1	[35]
*E. coli* O103:H2 str. 12009 DNA	86%	AP010958.1	[35, 36]
Phage VT2 phi_272	85%	HQ424691.1	-
*E. coli* O157:H7 str. TW14359	67%	CP001368.1	[37]
*E. coli* O157:H7 str. EC4115	67%	CP001164.1	[38]
*E. coli* O111:H- str. 11128	65%	AP010960.1	[36]
*E. coli* O157:H7 str. Sakai	65%	BA000007.2	[39]
*E. coli* Xuzhou21	65%	CP001925.1	[40]
*E. coli* O157:H7 EDL933	65%	AE005174.2	[41]
Stx2 converting phage II	64%	AP005154.1	[42]
Stx2 converting phage I	63%	AP004402.1	[43]
Enterobacteria phage Min27	63%	EU311208.1	[44]
Stx1 converting phage	60%	AP005153.1	[42]

The similarity score indicates the query coverage values with 98%-99% sequence identity. The hits with similarity values down to 60% are shown.

Recently, another EAHEC strain of serotype O111:H21 (strain 226), isolated from an HUS case occurred in Northern Ireland in 2012, has been described [13]. The short reads from the whole genome-sequencing project of this strain are available in NCBI sequence reads archive [NCBI SRA: SRA055981] and have been used for comparison. Unfortunately, among the 456 contigs obtained from the *de novo* assembly of the reads, the one including the *vtx2* genes was only 8,042 bp long, hindering the analysis of the entire sequence of the *vtx2*-encoding phage. Nevertheless, its whole-genome sequencing reads were aligned to the complete genomic sequence from the Phi-191 phage. The alignment was carried out with Bowtie2 software and visualized on the Integrative Genomics Viewer (IGV) free tool [45] (data not shown). This analysis failed to identify, in the genome of strain 226, a *vtx*2-phage with characteristics similar to that of Phi-191. In particular, the alignment showed that the reads from strain 226 mapped on the sequence of the Phi-191 phage only in the region comprising the Vtx2 A subunit-coding gene and extending 4.2 kb downstream the gene encoding the Vtx2 B subunit. It should be noted that alignments conducted using the whole short reads set could have generated a too high background noise deriving from the presence of sequences from other bacteriophages integrated in different regions of the chromosome of the O111:H21 strain 226. Nevertheless, the lack of sequencing reads aligning to the rest of the sequence of Phi-191 suggests that a different *vtx2* phage intervened in the emergence of this last EAHEC strain.

### Genomic comparison of Phi-191 with other vtx2-phages

In order to delve into the genomic organization of the different *vtx*2-phages we performed a progressive Mauve alignment between all the phages sequences used in the nucleotide comparison (Additional file 1: Figure S1). This analysis onfirmed the presence of similarities between the sequences but also highlighted that an extensive re-arrangement between the sequence-blocks must have occurred at some points during the phages’ evolution. This observation is in agreement with how reported in literature [46–48].

A deeper analysis of the Phi-191 genome was conducted by using phage sequences selected among those from typical VTEC displaying the highest similarity values (Table 1).

**Figure 3:**
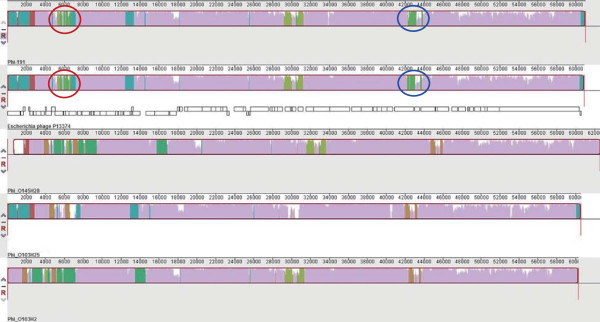
**Mauve Progressive Alignment of** ***vtx2*** **-phage genomes from EAHEC and VTEC strains showing the EAHEC-specific regions.** Blocks with the same colours indicate the *vtx2*-phages regions with identical DNA sequence. White blocks in a phage sequence indicate regions lacking of correspondence in the other sequences. Fragments of the phage genomes that are peculiar to EAHEC *vtx2*-phages are marked with circles: a red circle indicates the sequence encoding a hypothetical protein; the sequence encoding the phage fiber tail is encircled in blue.

The alignment of the Phi-191 sequence with those of phage TL-2011c (O103:H25), and the phages from the VTEC strains RM13514 (O145:H28) and 12009 (O103:H2), the causative agent of a romaine lettuce-associated outbreak occurred in the US in 2010 [35] and isolated in Japan from a sporadic case of diarrhoea [36], respectively, showed the presence of two sequence blocks that seemed to be peculiar to the EAHEC *vtx2*-phage and divergent from the same regions in the three classical VTEC associated-phages (Figure 3). One of the two regions was 1,500 bp long and comprised 140 bp of the 5’-terminus of a gene encoding a lysozyme (*rrrD*), two complete genes encoding the lysis protein S and a hypothetical protein, and 440 bp of the 3’-terminus of a gene annotated as a hypothetical protein. The 1,500 bp region corresponded to the nucleotidic positions 5,240-6,725 in the P13374 genome (ORFs 12–15). The other fragment was 900 bp long and spanned the region comprised between nucleotides 42,160 and 43,050 in the genome of the P13374 phage. The 900 bp fragment shared 100% homology with a region of 730 bp at the 3’-terminus of the ORF65 of P13374 phage encoding a phage tail fiber and a 72 bp fragment of the ORF66 coding for a tail fiber adesine.

### Distribution and analysis of the putative EAHEC-associated sequence regions amongst the vtx-phages

A BLAST comparison was conducted with the aim of investigating the distribution of the two putative EAHEC-associated regions among all the *vtx*-phage sequences available at NCBI. Such an analysis showed that only the 900 bp region encoding phage fiber tail was present in all the fully sequenced *vtx2*-phages from EAHEC strains, [GenBank:CP0032891.1; CP003301.1; CP003297.1] and divergent or absent in all the other phage sequences investigated. Accordingly, the presence of the sole sequence of 900 bp was assessed in the contigs derived from the *de novo* assembly of the short reads from the genome sequence of the O111:H21 strain 226 from Northern Ireland.

## Discussion

The *E. coli* genome continuously changes through both small-scale variations and horizontal gene transfer of mobile genetic elements (MGE). Among MGEs, bacteriophages play a pivotal role in the evolution of *E. coli* pathogenic clones [49] by providing a mean for genomic remodelling and conveying important virulence genes such as those encoding the Verocytotoxins (*vtx1* and *vtx2*) in the Verocytotoxin-producing *E. coli* (VTEC).

In 2011 a huge outbreak caused by an Enteroaggregative Haemorrhagic *E. coli* (EAHEC) O104:H4 struck Germany with more than 3,000 cases of infection, 800 HUS, and 50 deaths [12]. The causative agent was a mosaic strain deriving from the lysogenization of an Enteroaggregative *E. coli* strain with a *vtx2*-phage [8]. Such a virulence combination was indeed associated with elevate pathogenicity, as demonstrated by the high rate of human infections evolving to HUS, even among adults (88% and 42 years of median age), and the heavy toll of 50 deaths [6].

This arrangement of virulence factors in *E. coli* strains from human disease had been occasionally reported before during the investigation of a small outbreak of HUS occurred in France in 1992 and a case of infection in Japan in 1999 [10, 11].

The occurrence of the German outbreak caused the scientific community to look back retrospectively at the repositories of VTEC infections records and culture collections and it turned out that some other sporadic cases of infections with Vtx2-producing Enteroaggregative *E. coli* O104:H4 had already occurred in Europe and Asia in the time span 2001–2011 [12, 31]. Finally a HUS case occurred in Northern Ireland in 2012 was demonstrated to be associated with an EAHEC O111:H21 [13].

The observation of sporadic cases and outbreaks occurring throughout a 20-years time span, all caused by Vtx2-producing EAggEC and belonging to different serotypes, strengthens the hypothesis that these pathogenic *E. coli* represent a new pathogroup, as it has been recently proposed [9].

To better understand the events underlying the emergence of EAHEC we determined the whole genome sequence of Phi-191, the *vtx2-*phage present in the EAHEC O111:H2 isolated during the French outbreak of 1992, and compared it with the sequences of the *vtx2*-phage present in the EAHEC strains described in the following years and available in GenBank.

Interestingly, the genomic sequence of Phi-191 was almost identical to that of the *vtx2*-phages from the EAHEC O104:H4 strains isolated during the 2011 German outbreak about 20 years later. This is noteworthy since *vtx*-phages are characterized by a high degree of variability [50, 51]. It is conceivable that the same *vtx2*-phage has been acquired in two different events and that the selective pressure impeded the accumulation of changes in the phage sequence before the phage infection events occurred.

However, the EAHEC O111:H21 isolated in Northern Ireland in 2012 seems to host a different type of *vtx2*-phage, suggesting that at least two different *vtx2*-phage types have been successfully transferred to EAggEC. Unfortunately, the sequence of the phage of the EAHEC O86: HNM isolated in Japan in 1999 was not available for comparison [11].

It has been hypothesized that the infection with a lambdoid phage can be mediated by the cross-talking between the bacterium and the phage resulting in host specificity [52]. An extended comparison of the EAHEC *vtx*2-phages with the whole genome sequences of *vtx*-phages from VTEC strains available at NCBI returned a wide range of similarities between sequences, going from 87% to 60% and lower. This picture is in line with how reported for the general variability of *vtx*-phages sequences [50]. Interestingly, one region of 900 bp, identified in the Phi-191 and encoding a tail fiber, was present in all the *vtx*2-phages from EAHEC and was also present in the short reads dataset from the EAHEC O111:H21. At the same time this DNA sequence was absent in all the *vtx*-phage sequences identified in VTEC strains and stored at NCBI.

This is in agreement with previously reported data which pointed at a larger fragment including this region as one of those differentiating the P13374 genome from the *E. coli* phage TL-2011c (O103:H25) and not exhibiting significant homology to known *vtx*-encoding phages [31].

The annotation of the Phi-191 genome showed that this sequence peculiar to EAHEC *vtx2*-phages contains part of a gene encoding a type of phage tail fiber displaying some conserved aminoacidic motifs such as a Collagen triple helix repeat (20 copies) [NCBI CDD:189968] and the Peptidase_S74 [NCBI CDD:258151]. The latter is the C-terminal domain of the bacteriophage protein endosialidase, which forms homotrimeric molecules and releases itself from the end-tail-spike of the bacteriophages [53].

The 900 bp-long sequence could potentially encode part of the mechanism defining the specificity of the *vtx*2-phages for EAggEC strains, being directly involved in the phage-bacterium interaction. As a matter of fact, several authors reported that the interactions between phage tail fibers and host proteins, such as LamB and OmpC [52, 53] contribute to the success of the infection, as demonstrated by the finding that *lamB* gene mutations block phage adsorption [52]. It is therefore conceivable that differences in phage tail fibers may contribute to define *vtx2*-phages tropism for *E. coli* recipients, although this hypothesis together with the mechanisms underlying this process still need to be verified.

For a successful infection to occur, suitable *vtx*-phages and *E. coli* acceptors need to meet in the same environment. In the case of the emergence of typical VTEC, the events of *vtx*-phage acquisition probably occurred at the level of the gastrointestinal tract of ruminants [54] where both *vtx*-phages and aEPEC are abundant [55, 56].

Conversely, the EAHEC emergence is probably not directly connected to an animal reservoir since EAggEC are human pathogens with an inter-human transmission of the infection [1]. The environment, in turn, plays a role in the pathogen’s amplification cycles, particularly in geographic areas characterised by poor hygienic conditions, where the lack of effective human sewage treatments make the infections with enteric pathogens, including EAggEC, endemic [57]. In such a scenario, an environment contaminated with ruminant’s excreta might have been the source of the *vtx2*-phages found in EAHEC as it has been recently proposed [16]. Such a picture may account for the existence of a favourable setting for the EAggEC and the *vtx*-phages to come in contact and for the following selection process resulting in the occasional emergence of an *E. coli* strain matching the EAHEC definition.

## Conclusions

The *vtx2*-phages characterising EAHEC seem to belong to a sub-population of *vtx*-phages kept under selective pressure and characterised by the presence of a gene encoding a tail fiber, which could be involved in the mechanism used to recognize the EAggEC. The new EAHEC pathogroup may have emerged following multiple *vtx2*-phage acquisition events favoured by an overlapping of a human reservoir of pathogenic *E. coli*, the EAggEC, with the known animal reservoir of *vtx*-phages. The emergence of this new *E. coli* pathogroup further witnesses the great adaptability and plasticity of this bacterial species and underlines the need to rethink the global asset of hygienic practices to mitigate enteric infections worldwide; particularly in the presence of a global market of foodstuffs that is extending its boundaries towards low-income countries in the quest of new sources to meet the always increasing demand of cheap and exotic food commodities.

## Electronic supplementary material

Additional file 1: Figure S1: Mauve Progressive Alignment of Phi-191 genome with *vtx2*-phage genomes from EAHEC and VTEC strains showing the highest score of similarity. Blocks with the same colours indicate the *vtx2*-phages regions with identical DNA sequence. White fragments in a phage sequence indicate regions lacking of correspondence in the other sequences. Connecting lines link the same genomic block in different genomes and help to pinpoint the re-arrangement between *vtx*-phage genomes. (DOCX 435 KB)

## Abbreviations

aEPEC: Atypical enteropathogenic *Escherichia coli*
DEC: Diarrheagenic *Escherichia coli*
EAggEC: Enteroaggregative *Escherichia coli*
EAHEC: Enteroaggregative haemorrhagic *Escherichia coli*
HC: Haemorrhagic colitis
HUS: Haemolytic uremic syndrome
MGE: Mobile genetic elements
VTEC: Verocytotoxin-producing *Escherichia coli*
vtx: Verocytotoxin genes.
