# Supplementary figures and images for: Whole genome sequence comparison of vtx2-converting phages from Enteroaggregative Haemorrhagic Escherichia coli strains
Source: BMC Genomics. 2014 Jul 8;15(1):574. doi: 10.1186/1471-2164-15-574 (PMC4122784; doi:10.1186/1471-2164-15-574)

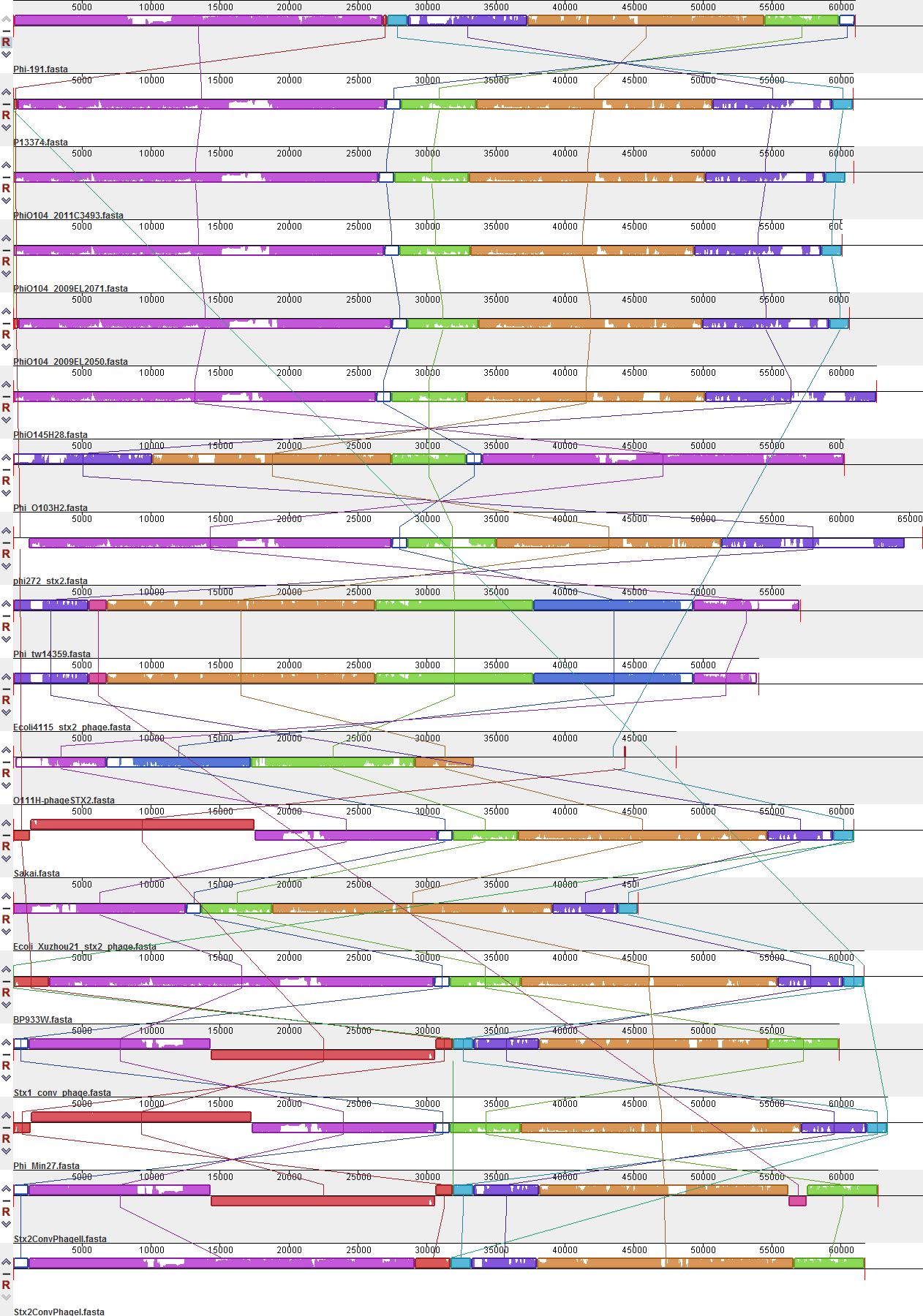

Supplement: Supplementary file 1 — Additional file 1: Figure S1: Mauve Progressive Alignment of Phi-191 genome with vtx2-phage genomes from EAHEC and VTEC strains showing the highest score of similarity. Blocks with the same colours indicate the vtx2-phages regions with identical DNA sequence. White fragments in a phage sequence indicate regions lacking of correspondence in the other sequences. Connecting lines link the same genomic block in different genomes and help to pinpoint the re-arrangement between vtx-phage genomes. (DOCX 435 KB) [file 12864_2014_6312_MOESM1_ESM.docx]
